# Clinical implementation of a commercial synthetic computed tomography solution for radiotherapy treatment of glioblastoma

**DOI:** 10.1016/j.phro.2024.100589

**Published:** 2024-05-17

**Authors:** Sevgi Emin, Elia Rossi, Elisabeth Myrvold Rooth, Torsten Dorniok, Mattias Hedman, Giovanna Gagliardi, Fernanda Villegas

**Affiliations:** a Department of Medical Radiation Physics and Nuclear Medicine, Karolinska University Hospital, 171 76 Stockholm, Sweden; b Department of Radiation Oncology, Karolinska University Hospital, 171 76 Stockholm, Sweden; c Department of Oncology-Pathology, Karolinska Institute, 171 77 Stockholm, Sweden

**Keywords:** MR-only, Glioblastoma, Radiotherapy, Synthetic-CT

## Abstract

**Background and Purpose:**

Magnetic resonance (MR)-only radiotherapy (RT) workflow eliminates uncertainties due to computed tomography (CT)-MR image registration, by using synthetic CT (sCT) images generated from MR. This study describes the clinical implementation process, from retrospective commissioning to prospective validation stage of a commercial artificial intelligence (AI)-based sCT product. Evaluation of the dosimetric performance of the sCT is presented, with emphasis on the impact of voxel size differences between image modalities.

**Materials and methods:**

sCT performance was assessed in glioblastoma RT planning. Dose differences for 30 patients in both commissioning and validation cohorts were calculated at various dose-volume-histogram (DVH) points for target and organs-at-risk (OAR). A gamma analysis was conducted on regridded image plans. Quality assurance (QA) guidelines were established based on commissioning phase results.

**Results:**

Mean dose difference to target structures was found to be within ± 0.7 % regardless of image resolution and cohort. OARs’ mean dose differences were within ± 1.3 % for plans calculated on regridded images for both cohorts, while differences were higher for plans with original voxel size, reaching up to −4.2 % for chiasma D2% in the commissioning cohort. Gamma passing rates for the brain structure using the criteria 1 %/1mm, 2 %/2mm and 3 %/3mm were 93.6 %/99.8 %/100 % and 96.6 %/99.9 %/100 % for commissioning and validation cohorts, respectively.

**Conclusions:**

Dosimetric outcomes in both commissioning and validation stages confirmed sCT’s equivalence to CT. The large patient cohort in this study aided in establishing a robust QA program for the MR-only workflow, now applied in glioblastoma RT at our center.

## 1 Introduction

Magnetic resonance (MR) imaging is used in brain radiotherapy (RT) planning to improve spatial accuracy due to its superior soft tissue contrast [1]. However, reported uncertainties arising from MR to computed tomography (CT) registration in brain range between 1–2 mm (depending on the registration algorithm used), leading to tumor underdosage due to their systematic nature [2], [3], [4], [5]. Deep learning (DL) algorithms capable of generating synthetic CT (sCT) images have proven to be clinically feasible as substitutes for CT, in terms of image quality and dose calculation capability [6], [7], [8], [9], [10]. Implementation of sCT in an MR-only workflow reduces the patient's radiation exposure, increases patient comfort, potentially reduces costs and improves resource allocation as CT would be excluded from the RT workflow [1], [11], [12], [13], [14], [15].

A debated issue with MR-Only RT is the geometric distortions intrinsic of MR imaging, caused by B_0_ inhomogeneity and non-linear gradients [16], [17]. Geometric uncertainties due to these distortions, can lead to dosimetric uncertainties when MR images are used for dose planning [18], therefore it is crucial to have a quality assurance (QA) program with clear tolerance criteria for these uncertainties, especially when sCT generated from MR is used for dose planning [13], [19]. As international QA guidelines for MR-only RT do not exist, local QA programs need to be adapted depending on the unique challenges that DL-based sCT presents.

This article presents an evaluation of the dosimetric performance of a commercial sCT for the brain in the context of clinical implementation of an MR-only workflow for glioblastoma RT treatment. Emphasis on the impact of voxel size on dose comparison is, for the first time, evaluated on a larger clinical cohort. To the best of our knowledge, only two other studies have published results from implementation of commercial DL-based sCT solutions for the brain [20], [21]. Therefore, the patient specific QA (PSQA) program developed and tested in clinical routine presented in this work will contribute to narrow the knowledge gap in PSQA procedures for MR-only implementation.

## 2 Material and Methods

### 2.1 Implementation process

Clinical implementation of the MR-Only workflow involved both retrospective and prospective studies conducted in three stages: commissioning, validation, and clinical use.

In the commissioning stage, sCT image- and dosimetric performance were retrospectively compared to the planning CT, while patients were treated through a routine CT-based MR-assisted workflow. Rossi et al*.* have presented an in-depth study on the assessment of image quality performance [22]. A QA program was then developed based on risks identified during the commissioning stage.

The validation stage aimed to test the QA program as patients were treated using the MR-only workflow, where a CT scan was still acquired as a back-up. The dosimetric performance of sCT in this stage was corroborated to the results from the previous stage.

Finally, as the CT was completely removed from the workflow during the clinical stage, the focus was to monitor the MR-only workflow and ensure that the communication chains were in place. Prior to each stage a risk analysis was performed involving representatives from various staff professions.

### 2.2 Patient selection

The commissioning stage included 112 adult patients diagnosed with CNS tumors with no MR contraindications and who provided informed oral and written consent. For the dosimetric analysis a sample of 30 glioblastoma patients were selected. During the validation stage, 48 patients diagnosed with grade IV glioblastoma and receiving photon treatment with the fractionation scheme provided in Table 1, were included. A retrospective dosimetric analysis was also performed for 30 of these patients. This study was approved by the Swedish Ethical Review Authority (dnr 2019–06404, with extension dnr 2023-03953-02).

**Table 1:** Patients included in the dosimetric analysis both during commissioning and validation stage. The diagnosis classification was done in accordance with WHO 2016 guidelines.

	Commissioning	Validation
Number of patients (*female/male*)	30 (*12/18*)	30 (*6/24*)
Diagnosis	Glioblastoma grade III without IDH-1 mutation, gliom grade IV	Glioblastoma grade IV
Median age (min *–* max)	58 years (*32*–*78*)	58 years (*38*–*75*)
Fractionation (*number of patients*)	3.4 Gy x 10 fr (*4*) 2.67 Gy x 15 fr (*7*) 2 Gy x 30 fr (*19*)	3.4 Gy x 10 fr (*6*) 2.67 Gy x 15 fr (*7*) 2 Gy x 30 fr (*17*)
Image for plan optimization	CT	sCT

### 2.3 Imaging and sCT generation

An individual neck support and a three-point thermoplastic mask was used as immobilization during both CT and MR examinations and during RT treatment sessions, following clinical routine. CT and MR examinations were done on the same day to minimize intra-modality variations. Both the CT- and MR-scanner were equipped with flat couch tops and external laser systems to reproduce the patient positioning at the linear accelerator. Prior to each scan, three markers were placed on the mask according to the laser intersection points, non-metallic CT markers (CT-23 from Suremark, Simi Valley, California, USA), and MR-compatible skin markers (PinPoint from Beekley Medical, Bristol, Connecticut, USA), respectively. CT images were acquired using a Siemens Somatom Definition AS + (Siemens, Erlangen, Germany), software version SynGo CT VB20A, with tube voltage 120 kV and 2 mm slice thickness with pixel size 0.98 x 0.98 mm^2^. MR images were acquired using a 3 T Philips Ingenia (Koninklijke Philips N.V., Best, The Netherlands), with software version R5.71 with RTgo 4.0. Flex and embedded posterior coils were used. T2-weighted, T1-weighted (with contrast medium) and fluid-attenuated inversion recovery (FLAIR) sequences were acquired for tumor and organs-at-risk (OARs) delineation. A 3D T1W mDixon sequence was acquired for generation of sCT using artificial intelligence (AI)-based Philips MR for calculating attenuation (MRCAT) Brain algorithm (v.4, Philips Oy, Vantaa, Finland). MR protocol details are provided in Table 2.

**Table 2:** Imaging protocols for the 3D T1W mDixon sequence for sCT generation and the sequence used for couch top detection.

	m**Dixon**	**Couch top detection**
Type	3D, T1-TFE	3D, TSE FLAIR
Scan time [min]	04:20	00:34
Number of slices	260	20
Slice thickness [mm]	1	0.75
Bandwidth/pixel [Hz/pixel]	868	871
Echo time [ms]	1.55 / 3.0	355
Repetition time [ms]	5.1	4800
TSE/TFE factor	30	200
3D distortion correction	3D	3D
Field of View [mm^3^]	232 × 270 × 260	198 × 250 × 15
Acquisition voxel size [mm^3^]	1.1 × 1.1 × 1.1	1.2 × 1.2 × 1.5
Acquisition matrix size [mm^3^]	211 × 244 × 236	165 × 208 × 10
Reconstructed voxel size [mm^3^]	0.68 × 0.68 × 1	0.52 × 0.52 × 0.75

#### 2.3.1 Couch top visualization

**Fig. 1:**
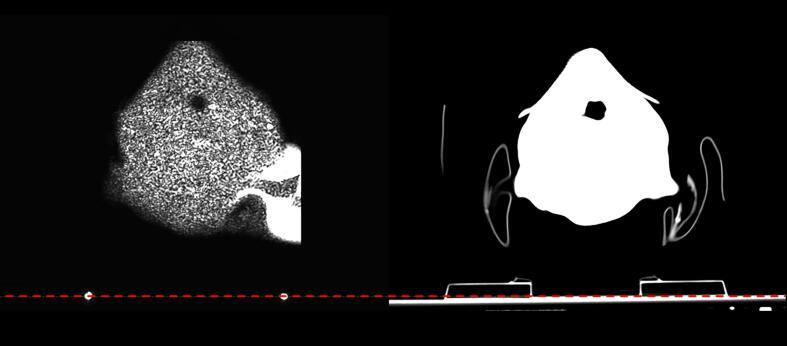
To the left, an image slice from TSE FLAIR MR sequence where the couch top markers can be seen. The corresponding CT image slice is shown to the right.

Standard MR sequences used in RT planning cannot visualize the couch top due to its attenuation properties. To address this, two MR-compatible skin markers were affixed to the couch top near the patient's immobilization device. A turbo spin echo (TSE) FLAIR sequence (protocol details in Table 2) with a large field-of-view was acquired over a small volume where these markers were positioned, see Fig. 1. These markers enabled accurate positioning of the couch top structure with predefined dimensions and Hounsfield Units (HU) in the treatment planning system (TPS). During validation, the method's feasibility was confirmed by comparing the couch structure's position to that on conventional CT images.

### 2.4 QA program

**Fig. 2:**
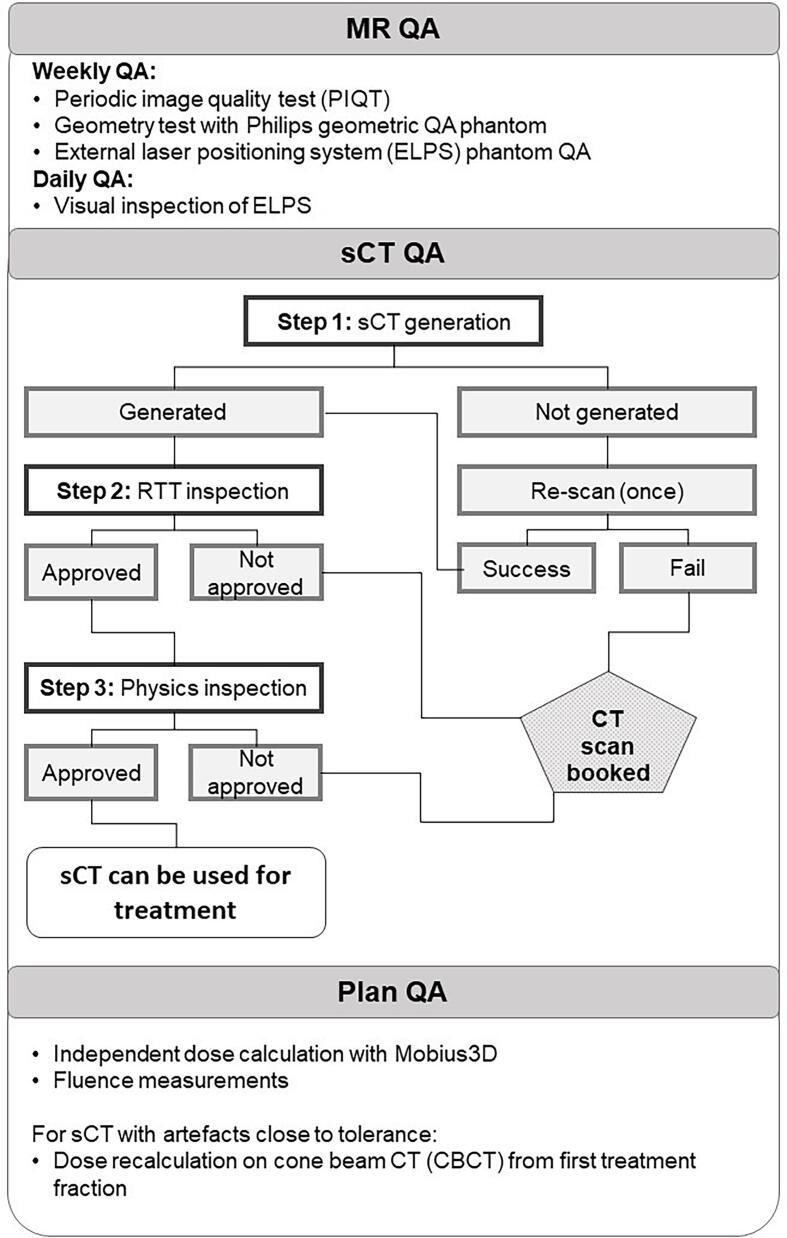
Patient specific QA workflow for MR-only RT. In this workflow, the MR QA follows vendor provided routines and phantoms. The sCT QA should be customized depending on the anatomical region and institutional routines. The plan QA is clinic specific.

The QA program adapted for MR-only RT is presented in Fig. 2.

#### 2.4.1 MR scanner QA

MR scanner QA is performed according to the vendor’s QA program. A periodic image quality test (PIQT) is performed weekly to monitor the quality of the MR images in the transversal plane. To evaluate geometric accuracy, a separate geometry test is also performed weekly using the Philips geometric QA phantom. Geometric distortion tolerance for a diameter of 20, 30, 40 and 50 cm from the scanner isocenter is 1.3, 1.8, 2.0 and 5.0 mm respectively. All analysis is performed using software provided by Philips. For the external laser positioning system (ELPS) a phantom QA is performed weekly, and a visual QA is performed daily.

#### 2.4.2 sCT QA

A patient specific QA (PSQA) program was developed to ensure that each patient’s sCT image quality was sufficient for dose planning, i.e., visual inspection of artefact occurrences (see Fig. 2). Following a dosimetric simulation for glioblastoma patients, a 3 cm size limit was set for sCT artefacts. The first checkpoint is performed by the software's internal sanity checks. If a sCT is generated, the second checkpoint consist of visual inspection by RT technologists (RTTs) directly at the MR scanner console for significant artefacts, such as missing body contour or hallucinations. If artefacts are within tolerance, the third checkpoint is performed by a medical physicist. Artefacts due to surgical fixation devices (SFDs) for cranial bone fixation, and bone resection gaps are evaluated in terms of size and position relative to the tumor. If found within set local tolerance values, then the sCT is approved for treatment planning. If sCT is not generated due to exceeded tolerance limits in the system's sanity check, or artefacts exceed tolerance level at any checkpoint, a CT scan is booked ideally within the hour of the MR scan.

#### 2.4.3 Treatment plan QA

Before delivering the treatment, a treatment plan QA is applied, including independent dose calculation using Mobius3D (v 4.0.1, Varian Medical Systems, Palo Alto, USA) and fluence measurements, following our standard clinical routine for any treatment with the volumetric arc therapy (VMAT) technique. For patients with artefacts close to the tolerance level and in the vicinity of the target, a dose recalculation is performed on the cone beam CT (CBCT) image from the first treatment fraction to evaluate appearance of hot- or cold-spots in the dose distribution.

### 2.5 Treatment planning and delivery

Treatment planning was performed in accordance with clinical routine and guidelines for OAR constraints, using 2 full arc, single isocenter VMAT technique with 6 MV in Eclipse TPS (v 15.6.5 and v 16.01.10, Varian Medical Systems, Palo Alto, CA, USA). The dose calculation algorithm AAA 15.5.12 was used during the commissioning stage, and AAA 16.1.00 during the validation stage. For conversion of HU to electron density, the Eclipse default calibration curve was preferred over the Philips provided MRCAT calibration curve due to likeness between the curves resulting in clinically insignificant differences. In daily practice choosing the default curve minimizes the risk of selecting the wrong calibration curve for treatment planning of diagnoses still treated with a conventional CT-based workflow.

The treatment was delivered on a Varian TrueBeam (v 2.7, Varian Medical Systems, Palo Alto, USA) linac. During the validation stage, a sCT-CBCT 6 degree-of-freedom image registration was performed every fraction prior to delivering the treatment.

### 2.6 Retrospective dosimetric analysis

For the retrospective dosimetric comparison, sCT and CT were regridded to the same image resolution using 3D Slicer (v 5.0.3, https://www.slicer.org/ [23]) and the image registration matrix exported from Eclipse. Target and OARs contours, as well as the clinical treatment plan, referred to as the reference plan in this work, were transferred to the rigidly registered images with original and regridded voxel sizes. For the commissioning cohort, the reference plans were optimized on CT, whilst for the validation cohort, optimization was performed on sCT. The recalculated plans on the original and regridded images will be referred to as test plans. A dose calculation resolution of 0.1 cm was used for all plans for the retrospective analysis.

The following dose-volume histogram (DVH) points were exported from Eclipse using visual scripting: mean dose (*D_mean_*) to clinical target volume (CTV), planning target volume (PTV) and left and right cochleae; *D2%* to PTV, brainstem and chiasm; and *D98%* to PTV. Dose differences were calculated using Equation (1), where *D_ref_* and *D_test_* represent the DVH points on to the reference and test plans, respectively.

(1) $$\Delta D=\frac{D_{\mathrm{ref}}-D_{\mathrm{test}}}{D_{\mathrm{ref}}}$$

### 2.7 Gamma analysis

MICE Toolkit (v. 2022.4.9, Nonpi Medical, Umeå, Sweden) was used to calculate gamma pass-rates between reference and test plans calculated on regridded images. The 3D global gamma index was calculated for brain and sCT body structures with a cut-off at 15 % of prescribed dose for the following dose/distance criteria: 1 %/1mm, 2 %/2mm and 3 %/3mm.

### 2.8 Statistical analysis

A statistical analysis comparing dose differences and gamma pass rates was performed in IBM SPSS Statistics (Version 29.0.1, Chicago). Commissioning and validation cohorts were also compared. For this comparison the absolute values were used, due to differences in the reference plans for the cohorts. A normality test was applied to the data and the choice of the statistical test was determined based on its results.

## 3 Results

### 3.1 sCT QA performance

Generation performance and image quality of sCT are presented in a previous work [22]. During the validation stage, there were no cases of failed sCT generation. One case failed the second check (presented in Fig. 2) as the tabletop markers were forgotten. Other 4 cases failed the third check due to either SFD artefacts or hallucinations exceeding the local tolerance level of 3 cm. These patients received treatment with conventional CT-based MR-assisted workflow.

### 3.2 DVH comparison

**Fig. 3:**
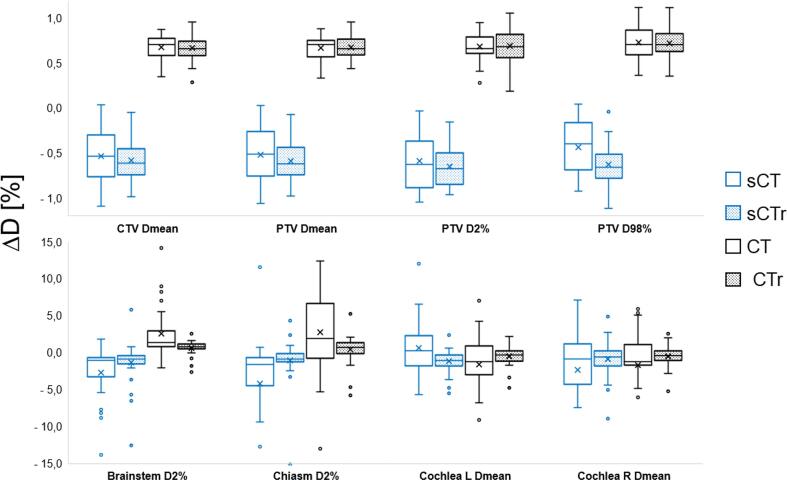
Relative dose differences (*ΔD*) between reference plan and test plans. During the commissioning stage (blue plots) the reference plan was optimized on conventional CT, whilst during the validation stage (black plots) the reference image was sCT. The dotted plots show the results from the comparison to plans calculated on images with regridded voxel size, whilst the remaining were calculated on the original image. Some outliers were excluded for OARs from the plot. (For interpretation of the references to colour in this figure legend, the reader is referred to the web version of this article.)

PTV and CTV mean relative dose differences were within ± 0.7 % regardless of image resolution and cohort as seen in Fig. 3. The OARs mean relative dose differences were within ± 1.3 % for plans calculated on regridded images for both cohorts, while the differences were higher for plans on original images, being up to −4.2 % for the chiasma *D2%* in the commissioning cohort.

For the validation cohort, there was no statistically significant dose difference between plans calculated on original and regridded images for the targets. For the commissioning cohort, a statistically significant difference was found for PTV *D_mean_* and *D98%*. When comparing cohorts, using plans calculated on regridded images, a statistically significant difference was found for CTV *D_mean_* and PTV *D_mean_* (see Table S1 in Supplementary Materials).

A larger variation in dose difference with more outliers was observed for OARs compared to targets. The variation was larger for plans calculated on original images, with an outlier (excluded from Fig. 3) at 46 % for right cochlea from the validation cohort. No statistically significant difference was found between the cohorts for OARs for plans calculated on regridded images. However, there was a statistically significant difference between plans calculated on original and regridded images for the *D2%* to the brainstem and chiasm in both cohorts.

Plans calculated on sCT yielded hotter plans than those calculated on CT, as shown by the positive target *ΔD* values from the validation cohort in Fig. 3, and the negative values of the commissioning cohort where the reference plan was calculated on CT.

### 3.3 Gamma analysis

Results from the gamma analysis are presented in Table 3. A statistically significant difference between the commissioning and validation cohorts for the different dose/distance criteria was found. Exceptions were the results for 2 %/2mm in sCT body structure and for 3 %/3mm in the brain structure. The gamma passing rate was lower for the sCT body compared to the brain and this difference was statistically significant (*p* < 0.001).

**Table 3:** Average gamma passing rate ± 1SD of 30 plans, both for the commissioning and validation stages, calculated on reference and regridded images. The results for the brain and sCT body are shown for three different dose/distance criteria with respective p-values from a Mann-Whitney *U* test comparing the commissioning and validation cohorts. The data marked with * was normally distributed.

	**Brain**			**sCT Body**		
	*Commissioning*	*Validation*	*p-value*	*Commissioning*	*Validation*	*p-value*
1 %/1mm	93.6 ± 7.6 %	96.6 ± 4.9 %	0.024	85.9 ± 6.9 %	91.3 ± 5.6 %	0.001
2 %/2mm	99.8 ± 0.4 %	99.9 ± 0.1 %	0.022	98.8 ± 0.8 %	99.0 ± 0.5 %*	0.391
3 %/3mm	100 ± 0.04 %	100 ± 0.1 %	0.115	99.9 ± 0.1 %	99.4 ± 0.3 %*	0.001

## 4 Discussion

This work presents an evaluation of the dosimetric performance of a commercial sCT for the brain for a larger clinical cohort with emphasis on the impact of voxel size on dose comparison. Moreover, development of a PSQA program, required for introducing the sCT into clinical workflow, is described.

Mean relative dose differences in previously published studies range between 0.1 % - 2.3 % to target volumes, and 0.1 % - 1.7 % to OARs [6], [20], [21], [24], [25], [26], [27], [28], [29]. Our results from commissioning and validation regridded images are found within agreement, even though the commissioning OARs presented higher dose differences than those in the validation stage. The observed decrease in dose difference and reduction in target dose variability of the validation cohort compared to the commissioning cohort is due to improved delineation guidelines introduced after the commissioning stage [22], and implementation of image quality control measures during the validation stage consisting of a clear guideline for image registration procedure standardizing the volume-of-interest used for image registration, which was previously lacking. Additionally, an independent control of the image registration performed by a medical physicist was introduced in the workflow. Decrease in dose difference was not observed for images with original voxel size, hence supporting the argument that regridding to same voxel size is necessary for fair dose comparison.

Large divergence in the relative dose difference remained for the OARs even after regridding. A possible explanation to the observed outliers in the regridded data is the effect of registration. Rigid image registration was used during the study to reflect clinical routine, making contour propagation from one image set to another susceptible to change. This effect was more evident for smaller structures, leading to larger differences in dose to cochlea and chiasm. *D2%* for the cohorts in this study were equivalent to 0.01 cc and 0.60 cc on average for the chiasm and brainstem, respectively. Hence, this measure was more sensitive to small changes in isodose lines, resulting in large variations in dose differences. The observed outliers for these OARs were in low dose regions and well under local dosimetric constraints. Therefore, the large relative dosimetric differences were not considered clinically relevant. Outliers may also be explained by the OARs proximity to bone-air cavities, which are known to yield similar signal in MR images, hence proving to be challenging for sCT generation [14].

The gamma analysis results were in agreement with previously published data [6], [9], [20], [21], [25], [26], [27], [28], [30], [31], [32], [33]. The gamma index was higher for the brain, than the body. Larger differences were observed near the body outline, likely due to registration uncertainties. The image registration procedure, introduced during the validation stage for transferring reference plan and structure set, led to improvement in the results for the gamma indexes.

Placing two markers on the couch top (see Fig. 1) was a reproducible way for determining the position of the couch, necessary for gantry clearance assessment while dose planning. The fact that the neck support is not visible on sCT may cause problems while troubleshooting patient positioning issues in treatment room (e.g., correct position of cervical vertebrae). A possible solution has been suggested by Masitho et al. [34], where silicon markers were used during MR scan to place a predefined auxiliary structure template for the immobilization device and the couch top during treatment planning. However, this method does not account for difference in setup for different patients.

SFD artefacts were observed in many patients, impacting target definition as described by Rossi et al. [22], and could account for some of the dosimetric differences observed in Fig. 3. These differences were not clinically relevant. The comparison between plans calculated on images with original and regridded voxel size, suggests that difference in voxel size has the largest impact on dose distribution.

The results of the image quality and dosimetric performance assessments demonstrated that the sCT is equivalent to the gold-standard CT when used in RT of glioblastoma and can be confidently integrated into clinical practice for this group of patients. However, more detailed evaluation (leading to perhaps tighter tolerance levels) of dose to smaller OARs, especially for those close to air cavities is warranted if the sCT solution is to be used for other clinical situations, where tighter dose constraints to OAR are required. Additionally, a solution for visualization of the patient immobilization (couch, mask, and neck support) in the sCT may be necessary if dose attenuation is to be considered in dose simulation.

Another limitation of this study is the part of the PSQA that is vendor specific (i.e. MR QA). However, the QA-program can be used as a template and be further adapted for other sCT solutions based on institutional routines. Additionally, for more accurate dose calculation on CBCT, for plan QA, image registration quality evaluation and CBCT dose calibration is necessary. The sCT-CBCT image registration accuracy during treatment sessions was qualitatively evaluated prior to delivering each treatment fraction by the RTTs, finding no difference to CT-CBCT matching. However, the quantitative analysis of the impact of sCT-CBCT positioning on geometric accuracy of MR-only workflow is left for future work.

To conclude, a commercial AI-based sCT solution for the brain was successfully implemented for glioblastoma patients with a well-functioning PSQA. Future work remains to implement a QA program for tracking the performance of the sCT solution over time and after software upgrades. The substantial size of the study cohort in this study lays a strong foundation for the development of international guidelines for benchmarking commercial sCT products.

## Credit authorship contribution statement

**Sevgi Emin:** Conceptualization, Methodology, Formal analysis, Writing – original draft. **Elia Rossi:** Writing – review & editing. **Elisabeth Myrvold Rooth:** Resources, Writing – review & editing. **Torsten Dorniok:** Software, Writing – review & editing. **Mattias Hedman:** Writing – review & editing, Project administration. **Giovanna Gagliardi:** Writing – review & editing. **Fernanda Villegas:** Conceptualization, Methodology, Writing – review & editing, Supervision, Project administration.

## Declaration of Competing Interest

The authors declare that they have no known competing financial interests or personal relationships that could have appeared to influence the work reported in this paper.
