# Supplementary material for: Clinical implementation of a commercial synthetic computed tomography solution for radiotherapy treatment of glioblastoma
Source: Phys Imaging Radiat Oncol. 2024 May 17;30:100589. doi: 10.1016/j.phro.2024.100589 (PMC11137592; doi:10.1016/j.phro.2024.100589)
Supplement: Supplementary Data 1 [file mmc1.pdf]

Supplementary Materials

Supplementary Table S1: The average dose difference for DVH metrics, with respective 5th- and 95th-percentile ranges, for both the commissioning and validation phases. Both sCT and CT had original voxel sizes, whilst sCTr and CTr had voxel sizes regridded to the resolution of the reference image. The reference image was CT during the commissioning stage and sCT during validation. The p-values from the statistical analysis comparing the dose differences between regridded and original plans are also presented for both cohorts. Additionally, the p-values comparing the dose differences in the regridded plans between the cohorts are presented for their respective DVH metric. T-tests were used for normally distributed data, marked with an asterisk (*). For the remaining data, the Wilcoxon test was used to compare doses on images with regridded and original voxel sizes, and the Mann-Whitney U test was used to compare cohorts.

DVH metric	*Commissioning*			*Validation*			*Cohort p-value*
	**sCT**	**sCTr**	**p-value**	**CT**	**CTr**	**p-value**	**Regridded**
CTV D_{mean}	-0.5% *(-0,9% - -0,1%)*	-0.6% *(-0.9% - -0.2%)*	0.064*	0.7% *(0.4% - 0.8%)*	0.7% *(0.4% -0.9%)*	0.864*	0.046*
PTV D_{mean}	-0.5% *(-0.9% - -0.1%)*	-0.6% *(-0.9% - -0.2%)*	0.013*	0.7% *(0.4% - 0.9%)*	0.7% *(0.4% - 0.9%)*	0.674*	0.044*
PTV D2%	-0.6% *(-1.0% - -0.1%)*	-0.7% *(-0.9% - -0.3%)*	0.068*	0.7% *(0.4% - 1.0%)*	0.7% *(0.5% - 1.0%)*	0.831*	0.154*
PTV D98%	-0.4% *(-0.9% - -0.1%)*	-0.6% *(-0.9% - -0.3%)*	0.000*	0.7% *(0.4% - 1.1%)*	0.7% *(0.5% - 1.0%)*	0.914*	0.086*
Brainstem D2%	-2.7% *(-11.3% - 0.9%)*	-1.3% *(-6.1% - 0.7%)*	0.005	2.5% *(-0.7% - 8.7%)*	0.6% *(-1.3% -1.6%)*	0.001	0.802
Chiasm D2%	-4.2% *(-17.1% - 0.6%)*	-1.1% *(-3.1% - 1.8%)*	0.002	2.8% *(-9.5% -17.7%)*	0.4% *(-3.3% -2.0%)*	0.026	0.751
Cochlea L D_{mean}	0.6% *(-4.0% - 6.1%)*	-1.1% *(-4.3% - 0.5%)*	0.088	-1.6% *(-8.1% - 3.5%)*	-0.4% *(-2.6% -1.7%)*	0.315	0.211
Cochlea R D_{mean}	-2.3% *(-13.1% - 4.8%)*	-0.9% *(-4.8% - 2.2%)*	0.347	-1.6% *(-5.5% - 5.7%)*	-0.5% *(-2.7% -1.8%)*	0.957	0.484
